# The efficacy of different interventions in the treatment of sarcopenia in middle-aged and elderly people: A network meta-analysis

**DOI:** 10.1097/MD.0000000000034254

**Published:** 2023-07-07

**Authors:** Qian Geng, Haiting Zhai, Liming Wang, Hongwen Wei, Shilun Hou

**Affiliations:** a Beijing Sport University, Beijing, China; b Naval Aviation University, Yantai, Shandong, China; c Qinghai Normal University, Qinghai, Xining, China.

**Keywords:** exercise, network meta-analysis, nutrition, physical performance, resistance, sarcopenia

## Abstract

In this study, a meta-analysis system was used to evaluate the effects of 7 methods of treating sarcopenia, including resistance exercise, aerobic exercise, mixed exercise, nutrition, resistance combined with nutrition, mixed exercise combined with nutrition, and electric stimulation combined with nutrition, and their effects on physical function.

**Methods:**

According to the PRISMA guidelines, PubMed, Web of Science, Embase, and other foreign databases, as well as Chinese databases such as China National Knowledge Infrastructure and Wan Fang, the literatures of randomized controlled trials with different intervention measures were searched. ADDIS software was used to compare and rank the results of the network meta-analysis.

**Results:**

A total of 2485 patients were included in the 30 randomized controlled trial items. According to the clinical manifestations of sarcopenia, 7 different forms of exercise and nutrition interventions can improve muscle strength, muscle mass, and physical function; in terms of improving muscle strength, resistance exercise has the most significant effect on improving grip strength (MD = 2.58, 95% confidence interval [CI] [1.06–4.07]); resistance exercise combined with nutrition lifting performed best in chair standing test (MD = −2.37, 95% CI [−4.73 to −0.33]). For muscle mass gains, resistance training increased appendicular skeletal muscle mass significantly (MD = 0.90, 95% CI [0.11–1.73]), while resistance exercise combined with nutrition significantly increased fat-free mass (MD = 5.15, 95% CI [0.91–9.43]). For physical activity, resistance training improved walk speed best (MD = 0.28, 95% CI [0.15–0.41]), and resistance exercise combined with nutrition in the best results were seen in the timed up and go test (MD = −2.31, 95% CI [−4.26 to −0.38]).

**Conclusion:**

Compared with aerobic exercise, mixed exercise, nutrition, resistance combined with nutrition, mixed exercise combined with nutrition, and electric stimulation combined with nutrition, resistance exercise has more advantages in improving muscle mass, strength, and physical function performance. The clinical treatment of sarcopenia with resistance exercise intervention has a better curative effect.

## 1. Introduction

Sarcopenia is a common progressive systemic muscle disease that commonly presents with decreased muscle strength, muscle mass, or reduced muscle physiology. According to recent analyses, the current prevalence of sarcopenia ranges from 8 to 36% in people under 60 years of age and from 10 to 27% in people over 60 years of age worldwide.^[1]^ The current proportion of aging is increasing worldwide, and it is predicted that the global population over 65 years of age will reach 426 million in 2050,^[2]^ and aging happens to be one of the main risk factors for sarcopenia.^[3]^ In addition, nutritional imbalance,^[4,5]^ systemic diseases,^[6,7]^ insufficient physical activity and poor lifestyle,^[8]^ and other risk factors may lead to the occurrence of sarcopenia.^[9,10]^ The adverse effects of sarcopenia on health should be recognized by more people. Because sarcopenia will not only increase the risk of falls and fractures,^[11–13]^ damage the ability to carry out activities of daily living and reduce the quality of life,^[14]^ but also affect the cognitive function of the elderly,^[15]^ increase the course of the disease, length of hospital stay and the cost of treatment,^[16–18]^ and even increase mortality.^[8,15,19]^ While sarcopenia seriously affects the quality of life of individuals, it also places a burden on the medical care of society as a whole.

So far, there is no specific medicine for sarcopenia. Starting from the risk factors of sarcopenia, it is considered that nutritional supplements and exercise intervention are more effective for sarcopenia^[3,20]^; however, there are different opinions about the effect of nutritional intervention on sarcopenia, which need to be further studied.^[21,22]^ Although exercise intervention has been proven to be effective in different studies in recent years, due to various types of exercise therapy, different scholars have been treating sarcopenia through different forms of exercise such as aerobic exercise, resistance exercise, and balance training. Different types of exercise have different forms of energy supply, resulting in different body adaptations, it is necessary to compare the intervention effects of different forms of exercise. In addition, there is no direct research comparison among some interventions, and the curative effect is still controversial. Therefore, it is necessary to integrate the current research on the treatment of sarcopenia, and compare the effects of different interventions through network meta-analysis,^[23]^ so as to provide reference for clinical selection of effective interventions.

## 2. Method

### 2.1. Search strategy and selection criteria

Through searching foreign databases such as PubMed, Web of Science, Embase, The Cochrane Library, and the randomized controlled trials (RCTs) in Chinese databases such as China National Knowledge Infrastructure and Wan Fang, the search time interval is from the establishment of the database to March 25, 2022. Chinese keywords are jishaozheng, kangzuyundong, youyangyundong, hunheyundong, dianciji, danbaizhi, yingyang, and English keywords are sarcopenia, aerobicexercise, resistance exercise, balance exercise, physical activity, protein, nutrition, supplement, acupuncture, whole-body vibration. The researchers choose a way to combine the corresponding subject words and free words. Two researchers (Z.H.T. and G.Q.) independently screened the literature, extracted information, and cross-checked it. Any disagreements are resolved through discussion or consultation with experts.

### 2.2. Literature inclusion criteria

Studies were considered to be eligible for inclusion according to the following criteria: RCTs for treating sarcopenia through resistance exercise, aerobic exercise, mixed exercise, nutrition, and electrical stimulation. The research subjects are patients with primary and secondary sarcopenia, regardless of gender or race. The research has detailed experimental design and steps; when the baseline values were consistent, the experimental group received different types of nutrition intervention and different forms of exercise intervention, and the intervention time was ≥8 weeks; the control group only received routine treatment, placebo (nutritional intervention) or health education. Finally, we excluded the literature with imprecise experimental design and steps, literature of review and animal experiments, studies that did not clearly provide the data needed for research, and literature that did not meet the inclusion criteria. For the repeatedly published literature, we selected the one with more complete data for inclusion.

### 2.3. Data extraction

Duplicate titles were excluded using Endnote´20 software (Endnote 20, Thomson ResearchSoft, American), for the published research data many times, we selected the one with the most complete data for inclusion. The database was created and full text was found, and the final included studies were extracted and entered by 2 searchers using an independent double-blind method for the relevant indicators. The data were extracted and entered as follows: authors and time of publication, age, sample size, intervention, intervention protocol (type of nutrition, duration of exercise, weekly exercise frequency, intervention period, etc), and outcome indicators. The outcome indicators extracted were: the appendicular skeletal muscle mass (ASMM), the fat-free mass (FFM), the grip strength, the walk speed, WS), the 5-chair stand test (CST), and the timed up and go (TUG).

### 2.4. Literature quality assessment

Two investigators (Z.H.T. and G.Q.) were independently double-blinded, and disagreements were resolved by discussion with experts. According to 7 items in Cochrane risk bias assessment tool, the quality of the included literatures were assessed.^[24]^ The assessment criteria were: random sequence generation method, hidden allocation scheme, blind method of subjects and researchers, blind method of evaluators, complete data of results, existence of selective reporting of research results, and other biases. Each entry was evaluated by “high risk of bias”, ”low risk of bias” and “unclear” (Fig. 2 and Table 1).

**Table 1:** Basic characteristics of included studies.

Inclusion research	Year of publication	Experimental group			Specific intervention	Control group			Intervention cycle/wk	Intervention duration	Outcome index
Author		Sample size	Age/years old	Intervention measure		Sample size	Age/years old	Intervention measure			
Kim et al^[25]^	2012	36	79.0 ± 2.9	Complex exercise	Warm up for 5 min, resistance for 30 min and gait balance for 20 min Upper limbs: pull down your arms and bend your biceps Lower limbs: ankle joint weight-bearing exercise, knee extension, hip flexion, and chair standing training	37	78.7 ± 2.8	No intervention	Twice/wk, 12 wk	60 min	①④
		34	79.5 ± 2.9	Complex exercise + nutrition		37	78.7 ± 2.8			60 min	
		37	79.2 ± 2.8	Nutrition	Essential amino acid	37	78.7 ± 2.8			N/A	
Kim et al^[26]^	2016	34	81.4 ± 4.3	Complex exercise	Aerobics: warm-up, fixed equipment training, power bicycle aerobics, chair standing training; Resistance: pull down your arms, bend your biceps, stretch your knees, and bend your hips Balance training and gait training	34	81.1 ± 5.1	No intervention	Twice/wk, 12 wk	60 min	①③④
		36	80.9 ± 4.2	Complex exercise + nutrition		34	81.1 ± 5.1			60 min	
		33	81.2 ± 4.9	Nutrition	Essential amino acids, catechins	34	81.1 ± 5.1			N/A	
Kim et al^[27]^	2012	30	79.6 ± 4.2	Complex exercise	Warm up for 5 min, resistance for 30 min, balance for 20 min, and gait training for 5 min to relax	28	80.2 ± 5.6	No intervention	Twice/wk, 12 wk	60 min	①③④⑥
		29	81.1 ± 3.7	Complex exercise + nutrition		28	80.2 ± 5.6			60 min	
		29	80.0 ± 4.0	Nutrition	Catechin	28	80.2 ± 5.6			N/A	
Liao et al^[28]^	2017	25	66.39 ± 4.49	Resistance	10 min warm-up and 40 min resistance	21	68.42 ± 5.86	No intervention	3 times/wk, 12 wk	45–50 min	②③④⑥
Cebrià et al^[29]^	2018	11	82.6 ± 9.1	Resistance	Warm up for 5 min, resist resistance for 20–30 min, and relax for 5 min Upper limbs: grip strength, wrist flexion, and extension, forearm pronation/supination, elbow flexion and extension, shoulder flexion/extension/adduction/abduction. Lower limbs: ankle flexion and extension, knee extension, hip flexion/abduction/adduction	17	81.2 ± 5.4	No intervention	3 times/wk, 12 wk	30–40 min	③④
Chen et al^[30]^	2017	15	69.3 ± 3.0	Aerobic	Step practice such as standing still, holding knees, lifting legs, rowing arms, swinging arms, twisting steps, lifting arms, squatting, V-step, etc	15	68.6 ± 3.1	No intervention	Twice/wk, 8 wk	60 min	③
		15	68.9 ± 4.4	Resistance	Upper limbs: shoulder push, biceps curl lift, triceps curl lift, bench press, standing rowing, unilateral rowing Lower limbs: hard pull, leg swing, deep squat, split squat	15	68.6 ± 3.1	No intervention		60 min	
		15	68.5 ± 2.7	Complex exercise	One aerobic training and 1 resistance training	15	68.6 ± 3.1	No intervention		60 min	
Maltais et al^[31]^	2016	8	64 ± 4.8	Resistance + nutrition	Upper limbs: bench press, shoulder push, bicep bend, rowing Lower limbs: leg lift, knee extension, and leg lift; core: sit-ups Nutrition: essential amino acids	10	64 ± 4.5	Resistance+ Placebo	Resistance 3 times/wk, 16 wk	60 min	⑤⑥
Yamada et al^[32]^	2019	28	84.7 ± 5.1	Resistance	Abdominal curl, hip flexion/extension/adduction/abduction, knee extension, ankle plantar flexion	28	83.9 ± 5.7	No intervention	Resistance Twice/wk, 12 wk	30 min	①③⑤
		28	84.9 ± 5.6	Resistance + nutrition		28	83.9 ± 5.7				
		28	83.2 ± 5.7	Nutrition	Nutrition: protein + vitamin D	28	83.9 ± 5.7			N/A	
Park et al^[33]^	2017	25	73.5 ± 7.1	Complex exercise	Resistance: elbow flexion, wrist flexion, shoulder lift, side lift, front lift, lying push, flying bird, side plate support, hard pull, squat, leg lift, ankle plantar flexion, etc. Aerobic: walking	25	74.7 ± 5.1	No intervention	5 times/wk, 24 wk	50–80 min	①③
Mason et al^[34]^	2013	117	57.9 ± 5	Aerobic	Aerobic: walking, power bike	87	57.9 ± 5	No intervention	5 times/wk, 12 wk	45 min	①
Dong et al^[35]^	2019	21	32.5～66.5	Resistance	Resistance: squeeze the elastic ball on the upper and lower limbs of 1 leg	20	50.5,～70.0	No intervention	3 times/wk, 12 wk	1–2 h	②③④
Hajj et al^[36]^	2019	60	73.05 ± 1.95	Nutrition	Nutrition: vitamin D	55	73.56 ± 2.14	Placebo	3 times/wk, 24 wk		①③
Zhu et al^[37]^	2018	36	74.8 ± 6.9	Complex exercise + nutrition	Nutrition: protein, vitamin D, β-hydroxyβ-methylbutyric acid, omega-3 fatty acid	37	72.2 ± 6.6	No intervention	Twice/wk, 12 wk	45–60 min	③④⑤
		40	74.5 ± 7.1	Complex exercise	Warm up for 5–10 min, resistance for 20–30 min, and aerobic for 20 min	37	72.2 ± 6.6	No intervention	3 times/wk, 12 wk		
Zdzieblik et al^[38]^	2015	26	72.2 ± 4.68	Resistance + nutrition	Nutrition: collagen peptide Resistance: pull down, leg lift, bench press, neck back press	27	72.2 ± 4.68	Resistance+ Placebo	3 times/wk, 12 wk	60 min	②
Vikberg et al^[39]^	2018	31	70.0 ± 0.28	Resistance	Resistance of lower limbs	34	70.0 ± 0.29	No intervention	10 wk	N/A	③⑤⑥
Tsekoura et al^[40]^	2018	18	74.56 ± 6.04	Complex exercise	Warm up for 5 min, resistance for 20–30 min, balance for 20 min, gait training and relaxation for 5–10 min	18	72.89 ± 8.31	Family education	Training Twice a week, 12 wk Walking 3 times/wk, 12 wk	Training for 60 min walking for 30–35 min	②③④⑤⑥
Piastra et al^[41]^	2018	33	69.9 ± 2.7	Resistance	Warm up for 5 min, resistance for 30 min, and relaxation for 15 min	33	70.0 ± 2.8	Posture training	Twice/wk, 36 wk	60 min	③
Dawson et al^[42]^	2018	13	68.6 ± 8.4	Resistance + nutrition	Upper limbs: chest push, shoulder push, sitting rowing, high pull-down Lower limbs: leg lift, leg bending lift, knee extension Core: flat support, hip bridge, dead bug Nutrition: protein	19	66.3 ± 9.0	Stretch+ Nutrition	3 times/wk, 12 wk	50 min	①②⑥
Chen et al^[43]^	2018	17	66.7 ± 5.3	Resistance	Kettlebell resistance: kettlebell swing, hard pull, goblet squat, lunge squat, rowing, 1 arm rowing, biceps curl, triceps stretch, double arm kettlebell push shoulder, Turkish rise, comprehensive dynamic training	16	68.3 ± 2.8	No intervention	Twice/wk 8 wk	60 min	①③
Mateo et al^[44]^	2014	49	70.8 ± 7.6	Nutrition	Whey cheese (IG/HD + RCH)	49	69.6 ± 6.4	No intervention	3 times/day, 12 wk	N/A	①③④⑤
Leheudre et al^[45]^	2007	12	58 ± 5	Nutrition	Isoflavone	6	58 ± 5	Placebo	24 wk	N/A	②
Bauer et al^[46]^	2015	139	77.3 ± 6.7	Nutrition	Vitamins, whey protein, total leucine	154	78.1 ± 7.0	Placebo	Twice a week, 12 wk	N/A	③④
Kenny et al^[47]^	2005	83	73.9 ± 0.6	Nutrition	Estradiol	84	74.7 ± 0.6	Placebo	12, 24, 36 mo	N/A	①⑤
Malafari et al^[48]^	2017	36	85.7 ± 6.5	Nutrition	Protein, fat, carbon, and water	38	84.7 ± 6.3	No intervention	42.3 ± 20.9 days	N/A	①②③④
Bo et al^[49]^	2017	30	73.23 ± 6.52	Nutrition	Whey protein, vitamin D, vitamin E	30	74.83 ± 5.94	Placebo	24 wk	N/A	①③⑤
Takeuchi et al^[50]^	2018	35	78.8 ± 5.1	Resistance + nutrition	Nutrition: branched-chain amino acids, vitamin D resistance: leg lift, leg bend lift, knee extension	33	80.9 ± 7.3	Resistance	8 wk	N/A	③
Dal Negro et al^[51]^	2010	16	75 ± 7	Nutrition	Essential amino acid	16	75 ± 7	Placebo	4 wk, 12 wk	N/A	②
Sammarco et al^[52]^	2017	9	53 ± 8.9	Nutrition	Protein	9	58 ± 10	Placebo	16 wk	N/A	②③
Nabuco et al^[53]^	2019	13	68 ± 4.2	Resistance + nutrition	Nutrition: whey protein Chest push, horizontal leg lift, sitting rowing, knee extension, free weight bicep bending lift, leg bending lift, triceps depression, sitting heel lifting	13	70.1 ± 3.9	Resistance+ Placebo	Nutrition Once a day, 12 wk Resistance 3 times/wk, 12 wk	N/A	⑤
Kemmler et al^[54]^	2018	24	77.1 ± 4.3	Electrical stimulation + nutrition	Nutrition: protein Electrical stimulation: WB-EMS	24	76.9 ± 5.1	No intervention	16 wk	N/A	①④

IG/HD + RCH = ricotta cheese, N/A = not available, WB-EMS = whole-body electromyostimulation.

Outcome index: ①ASMM = appendicular skeletal muscle mass; ②FFM = fat-free mass; ③Grip strength; ④Walk speed; ⑤CST = chair stand; ⑥TUG = timed up and go score.

**Figure 1.**
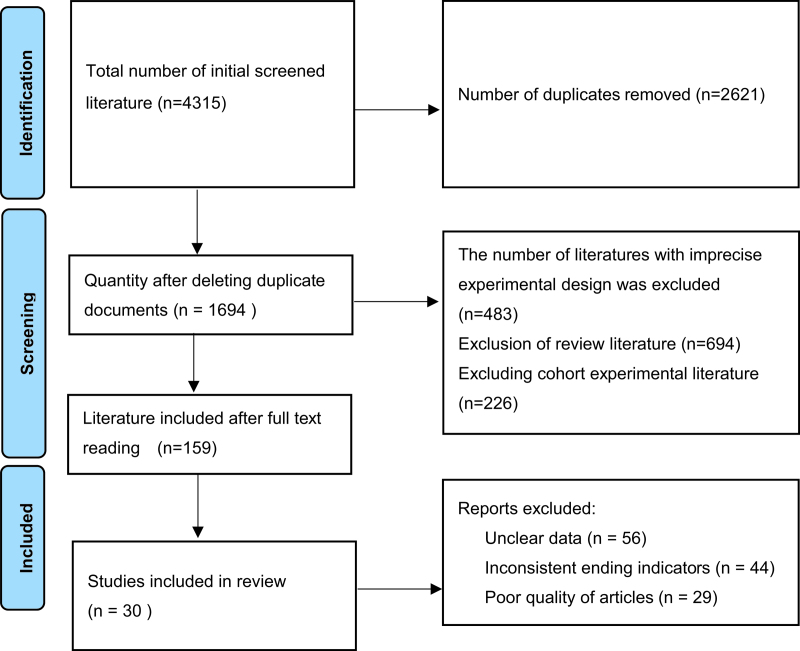
Flow diagram of study selection.

### 2.5. Statistical processing

The included literature was analyzed using the software of ADDIS 1.16.8 (Aggregate Data Drug Information System, Rotterdam) to determine network relationship maps and anecdotal ranking maps between the different interventions. The network meta-analysis of ADDIS was performed in a Bayesian framework, through the Markov chain-Monte Carlo algorithm. The included data were analyzed and processed (using 4 chains with an initial value of 2.5, a refinement iteration step of 10, an adjusted iteration number of 20,000, and a simulated iteration number of 100,000), with *P* < .05 and 95% confidence intervals (CI) as the basis for statistical differences, and the effect analysis statistic was expressed as the mean difference (MD), all effect sizes were expressed as 95% CI. The node-split model was used to test for inconsistency. The convergence of the model was evaluated by the potential scale-reduced factor.^[25]^ The results were corroborated by applying probability ranking plots to progress one.

## 3. Result

A total of 4315 documents were retrieved by searching various databases, including 3607 documents in English, 708 documents in Chinese, and 1694 documents remained after software and manual removal of duplicates, with reference to the inclusion and exclusion criteria, 159 papers were obtained by reading the abstracts and excluding non-conforming papers, 30 RCTs^[26–55]^ were finally included by further careful reading of the full text, and the literature screening flow diagram is shown in Figure 1.

**Figure 2.**
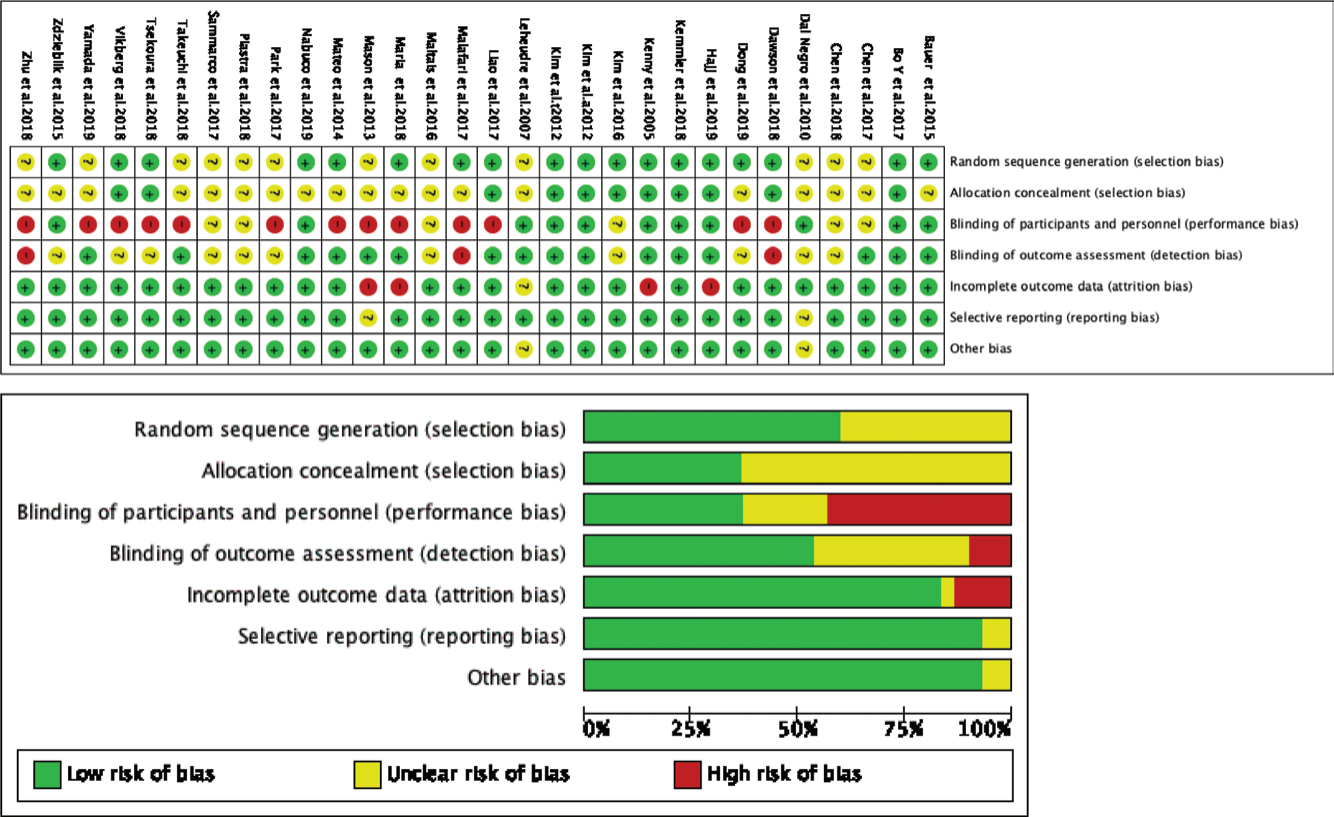
Diagram of the included literature with quality assessment.

A total of 2371 patients with sarcopenia were enrolled. Intervention measures in the experimental group included 8 resistance exercises, 2 aerobic exercises, 7 complex exercises, 13 nutrition, 6 resistance exercises combined with nutrition, 1 electric stimulation combined with nutrition, and 4 complex exercises combined with nutrition. Eighteen studies described the generation of random sequences in detail, while the remaining 12 studies did not specify. Table 1 shows the basic characteristics of the included study literature, and Figure 2 shows the results of the risk of bias assessment (Fig. 2).

**Figure 3.**
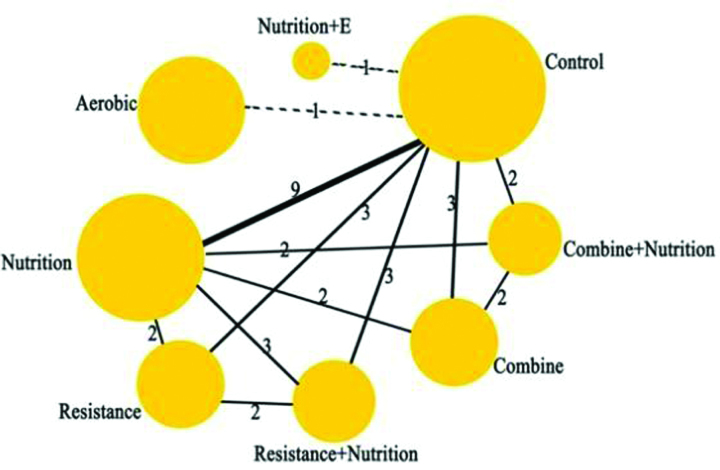
Network diagram between studies in ASMM. ASMM = appendicular skeletal muscle mass.

The 6 outcome indicators were analyzed separately for concordance. As potential scale reduced factor was 1, it suggested that the convergence was good, so the network meta-analysis was carried out under the consistency model. The network diagram of different intervention measures for myopathy is shown in Figure 3, with a total of 8 interventions including the control group. The numbers indicate the number of RCTs directly comparing the 2 interventions, and the circle size indicates the total number of participants in the different interventions. The larger the circle, the greater the number of participants. The connecting line indicates whether there is a direct comparison between interventions in the original study; the more direct comparisons between the 2, the thicker the connecting line; the absence of connecting lines indicates that there is no direct comparison between the original studies, and indirect comparisons can be made through network analysis (Fig. 3).

### 3.1. Results of network meta-analysis

#### 3.1.1. Muscle mass.

Muscle quality was examined by outcome indicators of ASMM and FFM. Fourteen studies^[26–28,33–35,37,43–45,48–50,55]^ reported the ASMM value and the decrease of muscle mass was the index to judge the muscular dystrophy.^[3]^ Taking MD as the effective quantity, Table 2 shows that among the 7 interventions, only resistance exercise (MD = 0.90, 95% CI [0.11–1.75]) has statistical significance compared with the control group. A pairwise comparison of 7 interventions shows that resistance exercise intervention is better than nutrition intervention (MD = 1.01, 95% CI [0.21–1.89]), and there is no statistical significance among other interventions. Nine studies^[29,36,39,41,43,46,49,52,53]^ reported FFM values, with MD as the effect quantity, table 2 shows that the intervention measures were compared with the control group. Among the 4 interventions, only resistance exercise combined with nutrition was statistically significant compared with the control group (MD = 5.15, 95% CI [0.91–9.43]). Pairwise comparison of the 4 interventions shows that resistance exercise combined with nutrition is better than resistance exercise alone (MD = −4.55, 95% CI [−8.60 to −0.49]), and there is no statistical significance among other interventions, as shown in Table 2.

**Table 2:** Results of the reticulated meta-analysis.

Measure 1 VS Measure 2		ASMM	FMM	Grip strength	Walk speed	Chair stand	TUG
Resistance exercise	Complex exercise	0.81 (−0.16, 1.89)	−1.30 (−6.51, 4.12)	0.19 (−1.79, 2.11)	0.15 (−0.02, 0.32)	−0.59 (−3.39, 2.11)	0.45 (−1.43, 2.28)
	Complex exercise + nutrition	0.86 (−0.19, 1.99)	–	1.18 (−1.03, 3.43)	0.19 (0.02–0.36)*	−1.39 (−4.30, 1.59)	0.08 (−2.12, 2.19)
	Nutritional intervention	1.01 (0.21–1.89)*	−1.18 (−4.53, 2.35)	1.58 (−0.31, 3.38)	0.24 (0.09–0.38)*	−1.29 (−3.88, 0.77)	−0.89 (−2.70, 1.03)
	Nutrition + electrical stimulation	0.37 (−1.36, 2.09)	–	–	0.25 (−0.09, 0.59)	–	–
	Resistance + nutrition	0.59 (−0.41, 1.61)	−4.55 (−8.60 to −0.49)*	0.05 (−2.57, 2.66)	0.10 (−0.11, 0.31)	0.11 (−1.27, 1.68)	0.57 (−1.21, 2.37)
	Aerobic	0.79 (−0.58, 2.22)	–	4.36 (−0.23, 9.00)	–	–	–
	Control group	0.90 (0.11–1.75)*	0.59 (−2.33, 3.60)	2.58 (1.06–4.07)*	0.28 (0.15–0.41)*	−2.26 (−4.40 to −0.42)*	−1.69 (−3.10 to −0.38)*
Complex exercise	Complex exercise + nutrition	0.03 (−0.80, 0.92)	–	1.01 (−0.82, 2.86)	0.04 (−0.09, 0.17)	−0.77 (−3.03, 1.57)	−0.36 (−2.20, 1.44)
	Nutritional intervention	0.18 (−0.54, 0.93)	0.10 (−4.66, 5.13)	1.39 (−0.29, 2.96)	0.09 (−0.04, 0.21)	−0.74 (−3.22, 1.31)	−1.34 (−3.02, 0.51)
	Nutrition + electrical stimulation	−0.46 (−2.14, 1.17)	–	–	0.10 (−0.23, 0.43)	–	–
	Resistance + nutrition	−0.22 (−1.35, 0.83)	−3.24 (−9.37, 2.91)	−0.13 (−3.13, 2.96)	−0.05 (−0.27, 0.17)	0.73 (−2.16, 3.56)	0.13 (−2.00, 2.33)
	Aerobic	−0.03 (−1.37, 1.29)	–	4.16 (−0.42, 8.76)	−	−	−
	Control group	0.08 (−0.61, 0.78)	1.86 (−2.51, 6.50)	2.40 (1.03–3.75)*	0.13 (0.02–0.24)*	−1.69 (−3.66, 0.14)	−2.12 (−3.59 to −0.77)*
Complex exercise + nutrition	Nutritional intervention	0.14 (−0.66, 0.96)	–	0.38 (−1.56, 2.25)	0.05 (−0.08, 0.17)	0.11 (−2.76, 2.28)	−0.95 (−2.86, 1.10)
	Nutrition + electrical stimulation	−0.51 (−2.24, 1.18)	–	–	0.06 (−0.27, 0.40)	–	–
	Resistance + nutrition	−0.26 (−1.45, 0.86)	–	−1.13 (−4.34, 2.08)	−0.09 (−0.31, 0.14)	1.51 (−1.61, 4.49)	0.52 (−1.87, 2.97)
	Aerobic	−0.07 (−1.44, 1.31)	–	3.15 (−1.66, 8.06)	–	–	–
	Control group	0.05 (−0.76, 0.81)	–	1.40 (−0.37, 3.09)	0.09 (−0.03, 0.21)	−0.88 (−3.23, 1.16)	−1.81 (−3.57, 0.07)
Nutritional intervention	Nutrition + electrical stimulation	−0.65 (−2.24, 0.91)	–	–	0.02 (−0.31, 0.34)	–	–
	Resistance + nutrition	−0.40 (−1.35, 0.44)	−3.39 (−7.61, 0.87)	−1.51 (−4.39, 1.45)	−0.13 (−0.34, 0.07)	1.40 (−0.81, 4.17)	1.45 (−0.44, 3.29)
	Aerobic	−0.21 (−1.46, 1.01)	–	2.77 (−1.89, 7.54)	–	–	–
	Control group	−0.10 (−0.59, 0.36)	1.76 (−0.35, 3.86)	1.01 (−0.17, 2.25)	0.04 (−0.05, 0.13)	−1.01 (−2.09, 0.50)	−0.83 (−2.53, 0.74)
Nutrition + electrical stimulation	Resistance + nutrition	0.24 (−1.53, 1.96)	–	–	−0.15 (−0.52, 0.21)	–	–
	Aerobic	0.44 (−1.43, 2.31)	–	–	–	–	–
	Control group	0.56 (−0.95, 2.05)	–	–	0.03 (−0.29, 0.34)	–	–
Resistance + nutrition	Aerobic	0.20 (−1.20, 1.69)	–	4.27 (−0.93, 9.49)	–	–	–
	Control group	0.31 (−0.56, 1.26)	5.15 (0.91–9.43)*	2.52 (−0.28, 5.30)	0.17 (−0.02, 0.38)	−2.37 (−4.73 to −0.33)*	−2.31 (−4.26 to −0.38)*
Aerobic	Control group	0.11 (−1.04, 1.24)	–	−1.78 (−6.32, 2.81)	–	–	–

TUG = timed up and go.

*P < 0.05.

#### 3.1.2. Muscle strength.

Muscle strength can be examined by changes in grip strength and outcomes of WS. Twenty studies^[26–28,30,31,33,34,36–38,40–42,44,45,47,49–51,53]^ have reported grip strength values. The grip strength test is easy to perform and widely used in clinical practice as a strength evaluation index for sarcopenia,^[15,56]^ with MD as the effector measure and Table 2 shows each intervention compared to the control group, respectively. Resistance exercise (MD = 2.58, 95% CI [1.06–4.07]) and complex exercise (MD = 2.40, 95% CI [1.03–3.75]) among the 6 interventions show that the grip strength is increased, which is statistically significant; Similarly, no other statistical differences were found. Eleven studies^[26–29,36,38,41,45,47,49,55]^ reported the WS. The WS test is safe and reliable, which can not only evaluate the strength of lower limbs but also be used as a powerful index to predict the deterioration of patients with sarcopenia.^[15,57]^ Taking MD as the effective quantity, Table 2 shows that each intervention measure is compared with the control group. Among the 6 interventions, resistance exercise (MD = 0.28, 95% CI [0.15–0.41]) and complex exercise (MD = 0.13, 95% CI [0.02–0.24]) increased the WS with statistical significance. Pairwise comparison of 6 interventions shows that resistance exercise is better than complex exercise combined with nutrition (MD = 0.19, 95% CI [0.02–0.36]), and better than nutrition intervention only (MD = 0.24, 95% CI [0.09–0.38]), as shown in Table 2.

#### 3.1.3. Functional performance.

Functional performance was judged by the time taken to complete the CST and TUG. Nine studies^[32,33,38,40,41,45,48,50,54]^ reported the CST time. The CST is a convenient and accurate test to assess lower limb muscle volume, strength, and functional performance^[15,58]^. Taking MD as the effective quantity, Table 2 shows each intervention separately compared to the control group. Among the 5 interventions, resistance exercise (MD = −2.26, 95% CI [−4.40 to −0.42]) and resistance combined with nutrition (MD = −2.37, 95% CI [−4.73 to −0.33]) increased the WS in a statistically significant manner. Six studies^[28,29,32,40,41,43]^ reported TUG time, TUG test is easy to operate and reliable in results. It can be used as an index to predict the risk of falling, and it is an effective method to evaluate physical activity^[15]^. Taking MD as the effective quantity, Table 2 shows each intervention measure compared with the control group. Among the 5 interventions, resistance exercise (MD = −1.69, 95% CI [−3.10 to −0.38]), complex exercise (MD = −2.12, 95% CI [−3.59 to −0.77]), resistance exercise combined with nutrition (MD = −2.31, 95% CI [−4.26 to −0.38]) had statistical differences. There is no statistical difference among other interventions, as shown in Tables 2 and 3.

**Table 3:** Ranking list of different interventions.

Intervention measure	ASMM	FMM	Grip strength	Walk speed	Chair stand	TUG score
Aerobic exercise	0.06	–	0.02	–	–	–
Complex exercise	0.01	0.12	0.26	0.02	0.22	0.34
Complex exercise + nutrition	0.02	–	0.04	0.01	0.06	0.12
Nutrition	0.00	0.03	0.00	0.00	0.02	0.01
Resistance exercise	0.55	0.01	0.30	0.77	0.29	0.07
Resistance + nutrition	0.05	0.85	0.39	0.14	0.42	0.46
Nutrition + electrical stimulation	0.30	–	–	0.06	–	–
Contrast	0.00	0.00	0.00	0.00	0.00	0.00

ASMM = appendicular skeletal muscle mass, TUG = timed up and go.

### 3.2. Probability ranking

**Figure 4.**
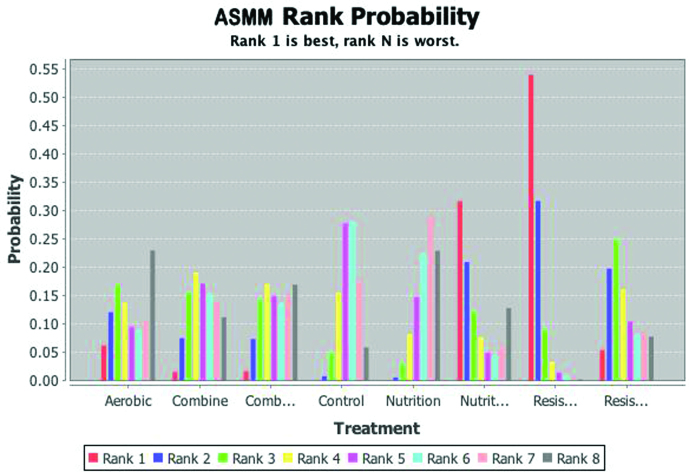
Ranking diagram of outcome indicators.

According to Bayesian statistical method, the intervention measures are sorted. The outcome indicators ASMM, FFM, grip strength, and WS are ranked with Rank1 as the best probability, using the Rank1 value as large as possible; CST and TUG are ranked with Rank6 as the best probability. The larger the Rank6 value, the better the intervention effect, as shown in Table 3 and Figure 4. Among them, the order of ASMM probability is resistance exercise (0.55) > nutrition + electrical stimulation (0.30) > aerobic exercise (0.06) > resistance exercise + nutrition (0.05) > complex exercise + nutrition (0.02) > complex exercise (0.01) > nutrition (0.00); The order of FFM probability is resistance exercise + nutrition (0.85) > complex exercise (0.12) > nutrition (0.03) > resistance exercise (0.01). The order of grip strength probability is resistance exercise + nutrition (0.39) > resistance exercise (0.30) > complex exercise (0.26) > complex exercise + nutrition (0.04) > aerobic exercise (0.02) > nutrition (0.00). The order of WS probability is resistance exercise (0.77) > resistance exercise + nutrition (0.14) > nutrition + electric stimulation (0.06) > complex exercise (0.02) > complex exercise + nutrition (0.01) > nutrition (0.00). The order of CST probability is resistance exercise + nutrition (0.42) > resistance exercise (0.29) > complex exercise (0.22) > complex exercise + nutrition (0.06) > nutrition (0.02). The order of TUG probability is resistance plus nutrition (0.46) > complex exercise (0.34) > complex exercise plus nutrition (0.12) > resistance exercise (0.07) > nutrition (0.01) (Fig. 4).

## 4. Discussion

In this study, the effects of 7 intervention measures on muscle mass, strength, and functional performance of patients with sarcopenia were compared for the first time through the network META-analysis, and the probability order of different intervention methods was given. Thirty literatures were included, and according to EWGSOP2^[15]^ in 2018, it was suggested to use grip strength and CST test to identify muscle strength, the results showed that resistance exercise and resistance exercise combined with nutrition had the best effect on improving strength level; TUG and WS are used to evaluate the performance of physical function, the results showed that resistance exercise was the best for improving WS, and resistance combined with nutrition improved TUG performance was the best; Finally, muscle mass was evaluated from physiological level by ASMM and FFM, the results showed that resistance exercise was the most effective way to increase ASMM, while resistance combined with nutritional intervention was the best way to improve FFM. The other intervention methods are superior to the control group with health education and routine treatment in the above aspects. This analysis shows that resistance training is more effective in improving muscle mass, muscle strength, and functional performance of patients with sarcopenia.

Sarcopenia originated in Greek and was first proposed in 1989 to describe the phenomenon of muscle atrophy and attenuation due to age.^[59]^ At first, it was considered a degenerative disease caused by aging, after clinical research, it was found that sarcopenia did not only occur in the elderly but also in young people with diseases.^[60–62]^ There are many causes of sarcopenia, according to the etiology, sarcopenia is divided into primary sarcopenia (muscle mass and strength decline caused by aging), and muscle mass begins to decline year by year after the age of 40^[15]^ and secondary sarcopenia (sarcopenia caused by systemic diseases such as cancer, malignant tumor, cardiovascular disease),^[10,62]^ decreased hormone secretion, insulin resistance, vitamin D deficiency, mitochondrial 3 dysfunction,^[9,63]^ as well as the decrease in the number and volume of muscle fibers^[64–66]^ and chronic inflammatory reaction caused by long-term oxidation of the body,^[67,68]^ the anabolic imbalance caused by the decrease of muscle protein synthesis rate^[69]^. At the same time, physical activity and insufficient nutrition intake are also important risk factors for sarcopenia.^[8,70]^ Muscle strength is an independent predictor and indicator of sarcopenia.^[71]^ In 2018, low muscle strength was listed as the main manifestation of muscular dystrophy in Europe and as a powerful clinical predictor, which is more conducive to early detection and early intervention. The reduction of muscle mass and strength is used as the basis for judging sarcopenia, while the severity of sarcopenia is defined by physical function performance.^[15]^ Therefore, the general survey should be conducted regularly through the a simple five-item questionnaire to prevent the aggravation of sarcopenia to the maximum extent.

At present, the analysis results show that resistance exercise and resistance combined with nutritional intervention have the best effect on relieving sarcopenia, and can effectively improve muscle mass, strength, and functional performance. No adverse events related to resistance exercise were reported in the study. Through the analysis of the mechanism of resistance exercise, the muscle contraction process is dominated by nerves, and the function of the nervous system will decline with age. The research of ROTH and SEIDLER et al^[72,73]^ shows that resistance exercise has a positive impact on nervous system, and resistance exercise can effectively activate nervous system, and accelerate muscle protein synthesis, thus increasing skeletal muscle mass and strength output. The research of Fragala et al^[74]^ shows that reasonable resistance exercise can improve the structural function of neuromuscular system, slow down the chronic inflammatory response caused by aging and regulate hormone secretion, thus stimulating muscle growth and remodeling, as well as improving muscle strength, muscle mass, and neuromuscular function. The research of Petriz et al^[75]^ shows that resistance exercise is more conducive to promoting the synthesis of muscle protein and increasing muscle mass. Resistance exercise is effective in increasing muscle strength and muscle mass for people of different ages. The form of exercise determines the form of energy supply and the adaptive changes produced by the body. Ferreira et al^[76]^ found that 40 to 65-year-old middle-aged and elderly people who want to improve their muscle strength must carry out resistance exercise; The meta-analysis of Grgic et al^[77]^ shows that resistance exercise is also effective for the improvement of muscle strength and quality of the elderly over 75 years old. In addition, compared with aerobic exercise, resistance exercise not only stimulates muscles but also exerts less pressure on the cardiopulmonary system, which is more suitable for older patients with sarcopenia. Resistance exercise has a greater gain on muscle strength, quality, and mobility than aerobic exercise and complex exercise, which is consistent with the conclusion of previous systematic review.^[78]^

Exercise intervention usually needs to reach a certain amount of training, duration, and load intensity^[10]^ to cause adaptive changes in the body. At the same time, it is necessary to ensure sufficient rest to prevent fatigue accumulation from causing damage. Among the resistance exercise intervention programs included in this study, 4 studies had exercise intervention twice a week,^[30,32,41,43]^ and 6 studies had exercise intervention 3 times a week,^[29,31,35,38,42,53]^ all of which lasted >8 weeks. During exercise intervention, it is necessary to ensure the recovery of the body while ensuring the training amount. Therefore, in the clinical intervention of resistance exercise, it is necessary to formulate the exercise prescription and nutrition plan in combination with the actual situation of patients and to carry out resistance exercise at least 2–3 times a week. Patients with myopathy accompanied by other diseases still need to be treated for related diseases during resistance and nutrition intervention. EWGSOP2 guide^[15]^ points out that those with high muscle content in youth have a low probability of developing sarcopenia, which reminds us that we should carry out exercise throughout the life cycle and continue to carry out health education at the same time. The research results of Tsekoura et al^[40]^ show that collective exercise with supervision and guidance in patients with sarcopenia is better than individual exercise in promoting muscle, bone, and other aspects due to the poor compliance of resistance exercise. Therefore, group resistance exercise intervention can be considered in clinics to increase interpersonal communication, so as to promote physical and mental health. When performing resistance exercise interventions for patients with sarcopenia, it is necessary to ensure proper exercise patterns as well as the gradual progress of resistance loads and exercise difficulty. Fixed equipment and elastic bands are commonly used for resistance exercise in patients with sarcopenia in the current study.

The literature included in this analysis conducted nutritional intervention for patients with sarcopenia by supplementing relevant nutrients conducive to muscle synthesis^[27,36,46]^. Hormones and essential amino acids^[25,26,31,45,47,50,51]^ that promote anabolism of protein, and direct supplementation of protein^[32,38,42,44,48,49,52–54]^ intervened in the nutrition of patients with sarcopenia. The above nutrition ultimately promotes muscle synthesis through protein supplementation.^[79]^ However, the results of our analysis showed that sarcopenia intervention through nutritional supplementation alone is not effective. As the body ages, muscle protein synthesis decreases in the body, and researchers hypothesized that consuming more protein would help promote muscle protein synthesis. A meta-analysis by Gielend et al^[80]^ in 2021 compared different nutrients and found that leucine was more effective for sarcopenia relative to other nutrients while suggesting the combination of resistance exercise during clinical interventions. While Remelli et al^[81]^ showed that vitamin D supplementation was effective in slowing down sarcopenia. However, Studenski et al^[82]^ showed that due to anabolic resistance, even if patients with sarcopenia ingest sufficient protein, the effect of muscle enhancement is not obvious in the case of low muscle synthesis sensitivity. Consistent with the results of this paper, the review of Beaudart et al^[21]^ showed that the effect of nutritional intervention alone is very limited, especially for patients who do not lack nutrition. It can be concluded that the effect of nutritional intervention on patients with sarcopenia depends on whether the patients themselves lack some nutrition. Supplementing the nutrition they lack is only the most basic condition, and it is also necessary to promote the development of muscle and physical activity through resistance exercise.

## 5. Study strengths and limitations

There are some limitations to our review. First, the number of studies with different interventions included in this paper varied widely (e.g. 13 for nutritional interventions and 2 for aerobic interventions). Second, some of the literature failed to describe the randomization and allocation process of the experiments in detail, which may cause bias in the results. Finally, the literature involved a wide variety of training methods, and the analysis results showed that the effect of resistance exercise was optimal. The specific quantitative-effective relationship of resistance exercise could not be clarified because the literature involved different forms of resistance and did not reach complete agreement in terms of training load and intervention time.

For the future treatment of sarcopenia, there are many new training tools available today, such as blood flow restriction compression training, which have been shown to produce greater training gains with a smaller training load. Therefore, after it is clear that resistance exercise is more effective, more efficient training methods including compression resistance can be further investigated. In addition, resistance exercise for patients with sarcopenia is currently mainly based on upper and lower extremity strength training, and core stability helps prevent falls. Since only a small number of studies involved follow-up results, future studies should investigate follow-up results in order to clarify the long-term effects of treatment measures. Finally, patients with sarcopenia often have >1 chronic disease and commonly suffer from other conditions, so individualized exercise prescriptions are recommended when performing resistance exercise interventions.

## 6. Conclusions

To sum up, through network meta-analysis, this review determined that resistance exercise has more advantages in muscle mass, strength, and physical function performance than aerobic exercise, mixed exercise, nutrition, resistance combined nutrition, mixed exercise combined nutrition, and electrical stimulation combined nutrition, which can provide a basis for resistance exercise intervention in middle-aged and elderly patients with sarcopenia.

## Author contributions

**Conceptualization:** Qian Geng, Hongwen Wei.

**Data curation:** Haiting Zhai.

**Formal analysis:** Qian Geng, Haiting Zhai.

**Funding acquisition:** Hongwen Wei.

**Investigation:** Haiting Zhai.

**Methodology:** Qian Geng, Shilun Hou, Liming Wang.

**Project administration:** Hongwen Wei, Shilun Hou.

**Resources:** Hongwen Wei, Shilun Hou.

**Software:** Qian Geng, Liming Wang.

**Supervision:** Haiting Zhai, Hongwen Wei, Shilun Hou.

**Validation:** Qian Geng, Haiting Zhai.

**Visualization:** Qian Geng.

**Writing – original draft:** Qian Geng.

**Writing – review & editing:** Haiting Zhai, Hongwen Wei.
